# *Crinum bulbispermum*, a Medicinal Geophyte with Phytostabilization Properties in Metal-Enriched Mine Tailings

**DOI:** 10.3390/plants13010079

**Published:** 2023-12-26

**Authors:** Vincent C. Clarke, João Marcelo-Silva, Sarina Claassens, Stefan J. Siebert

**Affiliations:** 1 Unit for Environmental Sciences and Management, North-West University, Potchefstroom 2531, South Africa; charl.clarke@nwu.ac.za (V.C.C.); sarina.claassens@curtin.edu.au (S.C.); 2 School of Molecular and Life Sciences, Curtin University, Bentley, WA 6102, Australia

**Keywords:** bioaccumulation, geophyte, grasslands, potentially toxic metals, restoration

## Abstract

Ancient grasslands are lost through transformation to agriculture, mining, and urban expansion. Land-use change leads to ecosystem degradation and a subsequent loss of biodiversity. Globally, degraded grasslands have become a priority for restoration efforts to recover lost ecosystem services. Although the ecological and social benefits of woody species and grasses are well documented, limited research has considered the use of forbs for restoration purposes despite their benefits (e.g., C sequestration and medicinal uses). The aim of this study was to determine if *Crinum bulbispermum* (Burm.f.) Milne-Redh. & Schweick., a medicinal geophyte, could form part of restoration initiatives to restore mine soils in grasslands of the South African Highveld. A pot experiment was conducted to assess the performance of *C. bulbispermum* in a random design, with three soil treatments varying in level of degradation and metal contamination. The plants were monitored for 12 months, and the morphological characters were measured monthly to assess performance and survival. Inductively coupled plasma mass spectrometry (ICP-MS) was used to determine the soil and plant tissue concentration of potentially toxic metals. The results indicated that mine tailings negatively affected the growth and development of *C. bulbispermum*. Although the survival rates indicated that it could survive on tailings, its below-par productivity indicated that the species is not ideal for restoration purposes unless the tailings are ameliorated with topsoil. Although there was root accumulation of metals (Co, Cd, Cu, Mo, and Zn), there was no translocation to the bulbs and leaves, which makes *C. bulbispermum* suitable for medicinal use even when grown on metal-enriched soil. This species may not be viable for phytoremediation but is a contender to be used in phytostabilization due to its ecological advantages and the fact that it does not accumulate or store metals. These findings underscore the importance of considering geophytes in grassland restoration strategies, expanding their ecological and societal benefits beyond conventional approaches.

## 1. Introduction

Grasslands cover about 40% of the world’s surface and provide many beneficial ecosystem services [1]. In South Africa, the grassland biome is one of the most threatened biomes with a 35% transformation rate [2]. Despite its ecosystem importance, grasslands are being degraded and transformed by agriculture and mining activities [3]. Mining is a critical aspect of the economy but is accompanied by many industrial activities with a wide range of negative impacts on the environment, including the contamination of surface and groundwater, soil degradation, and loss of biodiversity and the potential for future land use [3]. Mining activities increase the geographical footprint of degraded areas and include those areas occupied by and surrounding loose soil piles, bare-stripped areas, waste rock piles, and subsided land areas. Open-pit or surface mines cause more land-use damage than traditional-style shaft mines due to their much larger footprint. Many fertile land areas have been irreparably degraded due to mining and its related activities [4,5]. Environmental contamination due to potentially toxic elements is an ecotoxicological threat originating from mining-related industries and is non-biodegradable and persistent in nature, causing soil and water pollution [6]. Smelting processes are key sources of potentially toxic metal(loids) (PTMs) in water and soil [7]. Although these industrial processes are necessary for producing and extracting metals for economic purposes, contamination by PTMs can lead to toxicity at certain concentrations [8]. Tailings from gold mines contain liquid and solid waste, with high concentrations of toxic elements such as As, Cd, Cu, Cr, and Pb [9]. A study identified As, Co, Cr, Pb, and Se as the most common elements in the soil around platinum mines, combined with low pH, which can impact the growth of plants negatively [10].

Plants require low concentrations of micronutrients, including some PTMs such as Cu, Cr, and Zn, to ensure their growth and upkeep [11]. However, when the concentration of these micronutrients increases beyond a certain threshold in plant tissue, it becomes toxic and limits plant growth and development [11,12]. Soil pH and soil structure can also influence nutrient toxicity; highly acidic soils can lead to Al and Mn toxicity and a deficiency in Mo. In comparison, alkaline soils result in B toxicity and Fe, Mn, and Zn deficiency [11]. As PTMs cannot be used or broken down by a plant during excessive uptake, their accumulation negatively affects plant growth and development [13]. The most common effects caused by toxic concentrations of PTMs are the inhibition of cytoplasmic enzymes and the damage to cell structures due to oxidative stress [14].

Despite the well-documented potential toxic effects to plants, phytoremediation is a common practice, utilizing plants to clean up and revegetate contaminated sites [15]. A variety of techniques with different applications are included under the term phytoremediation. Plants can either remove, immobilize, or degrade contaminants [15]. The process whereby plants remove organic and inorganic compounds from their environment (soil and water) is called phytoextraction, phytofiltration, or rhizoextraction [16]. Conversely, phytostabilization is a process applied in restoration whereby inorganic contaminants (e.g., metal(loid)s) are immobilized in the soil, minimizing their transport in dust or water [16,17]. The plants used for phytoremediation are considered in two categories, namely, metal hyperaccumulators and excluders. Hyperaccumulators are usually forbs that take up PTMs within their living tissue at levels up to thousands of times greater than those for other plants [18,19]. Excluders are plants that limit the translocation of certain metals and store very low levels of metals in their tissue, but they can still contain relatively higher amounts of metals in their roots [20]. Phytoremediation has major potential to restore contaminated land by contributing to the regeneration of lost natural processes and ecosystem services [21].

The importance of forbs for restoration purposes has long been neglected, despite forbs providing numerous ecosystem services [22], having medicinal qualities [23], providing forage for livestock [24], contributing to carbon sequestration [25], playing host to beneficial insects [26], and acting as grazing indicators [26]. Globally, initiatives are underway to better understand the importance of forbs within the ecosystem [24] and incorporate them into restoration plans [27]. The potential of forbs in restoration is undocumented, despite the very large diversity of this life form in the ecosystem. In South African grasslands, forb species in a plant community outnumber grass species 5:1 [22]. Nonetheless, grass species remain the preferred choice for restoration efforts as their cultivation requirements are well-understood [28].

The aim of this study was to determine if a geophytic forb with a large underground storage organ with medicinal use, *Crinum bulbispermum*, can be used in the restoration processes of mine tailings. We tested whether the potential toxicity of the tailings would affect plant growth (ability to provide ecosystem services) and leaf toxicity (risk to consumption for medicinal purposes). The objectives were to assess plant survival and performance in different degraded soils, and determine whether plants take up and store PTMs in their organs.

## 2. Results

The tailings were nutrient-poor compared to the soil from the control treatment (Table 1). There was a clear difference between the three soil treatments, with the control soil having the lowest pH and the tailings having the expected higher levels of PTMs (Table 1). This confirmed a PTM enrichment gradient from the control, which was the least metalliferous, to the platinum tailings as the most metalliferous (highest concentrations of PTMs).

**Figure 1:**
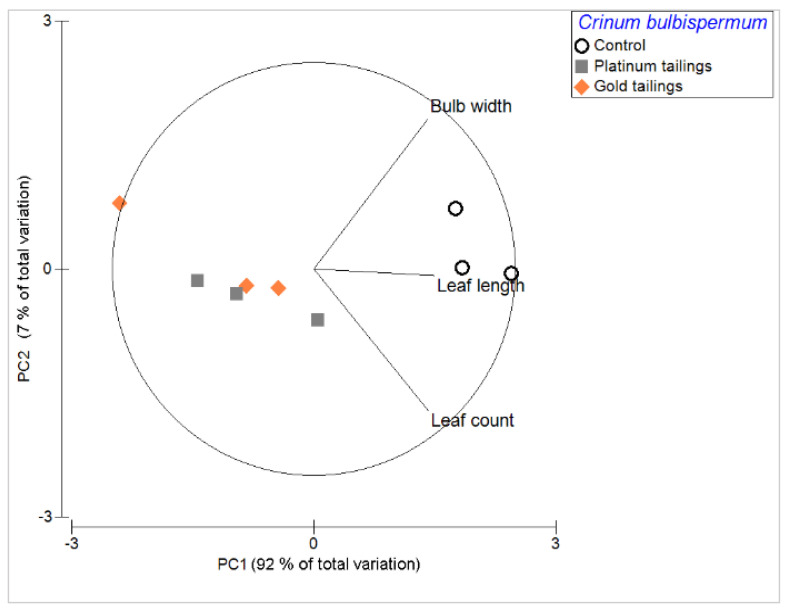
Principal component (PC) analysis with plant developmental variables for *C. bulbispermum* across the treatments.

**Figure 2:**
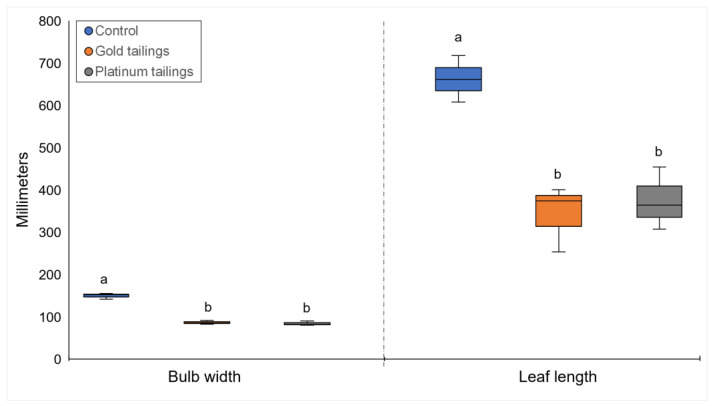
Developmental variables with significant differences for *C. bulbispermum* among the treatments. Different letters indicate a significant difference (*p* < 0.05) between treatments within a single variable.

**Figure 3:**
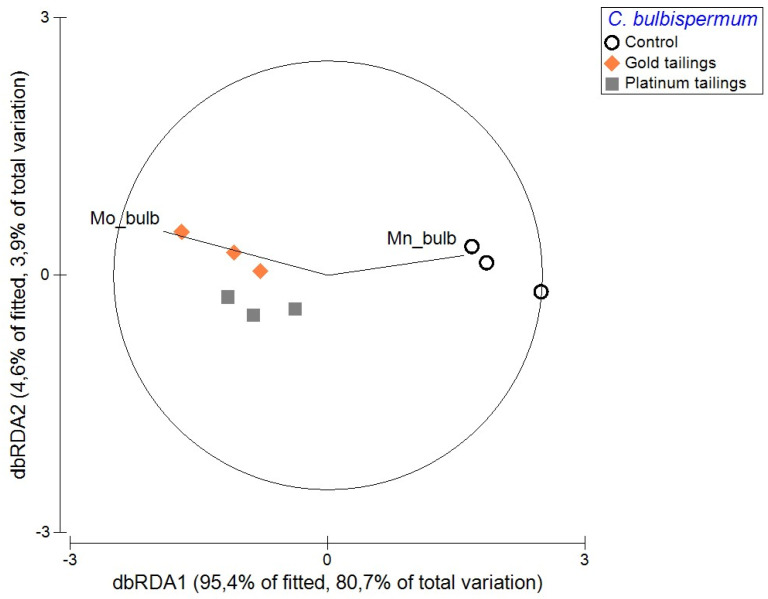
Distance-based redundancy analysis (dbRDA) plot with the main predictive variables (vectors of PTMs from study organs) on bulb development.

Enhanced growth of bulbs, longer leaves, and higher number of leaves per plant were associated with the control soil and not with the mine tailings (Figure 1). *Crinum bulbispermum* survived in both tailings treatments but had significantly (*p* < 0.05) shorter leaves and smaller bulbs compared to the control treatment (Figure 2). Specific elements were associated with the affected development and growth of *C. bulbispermum* bulbs (Figure 3). Higher concentrations of Mo (in the tailings) were associated with smaller bulbs (width), and higher concentrations of Mn (control) were associated with larger bulbs (width). The concentration of Mo was higher in the organs from the tailings compared to the control, even though the concentration of Mo in the control soil was similar. The inductively coupled plasma mass spectrometry (ICP-MS) analysis of the soils also showed that the concentration of Mn was highest in the platinum tailings and lowest in the gold tailings.

**Figure 4:**
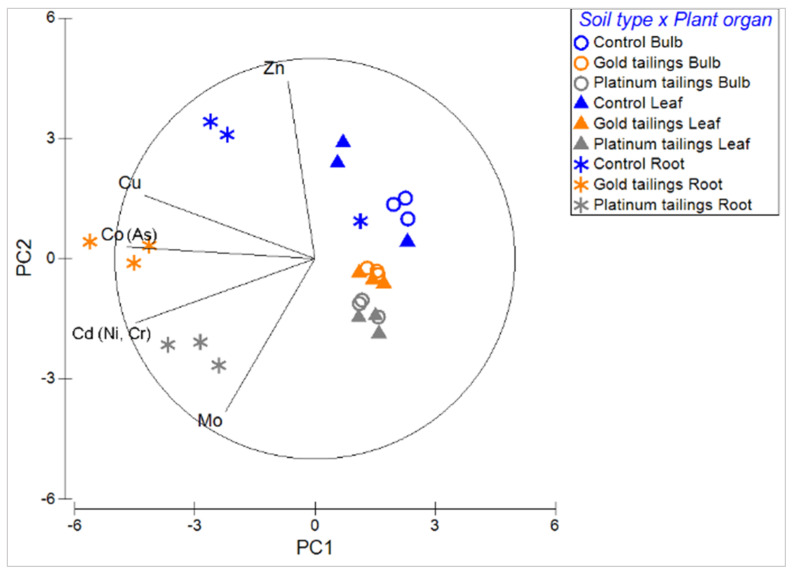
Principal component (PC) analysis with the main PTMs (Cd, Co, Cu, Mo, and Zn) as vectors associated with concentrations in plant organs across the treatments. Elements within brackets are highly correlated (r > 0.9), with the main element listed before the brackets.

**Figure 5:**
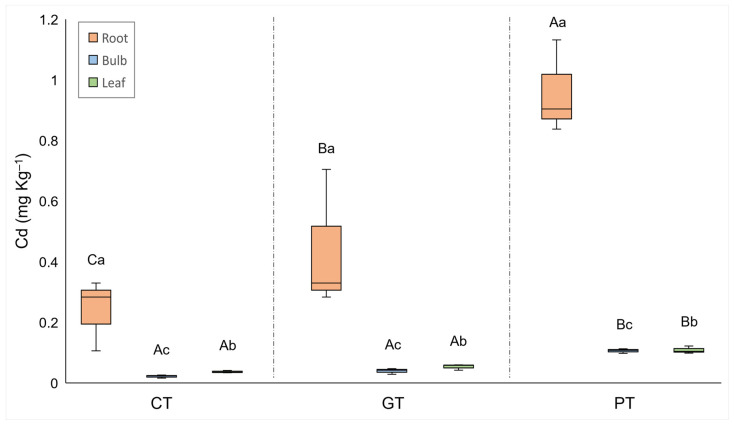
Median, quartiles, and range of Cd concentrations in different plant organs. Treatments: CT = control, GT = gold tailings, and PT = platinum tailings. Different capital letters indicate a significant difference among treatments for the same organ (*p* < 0.05); different small letters indicate a significant difference among organs within the same treatment (*p* < 0.05).

**Figure 6:**
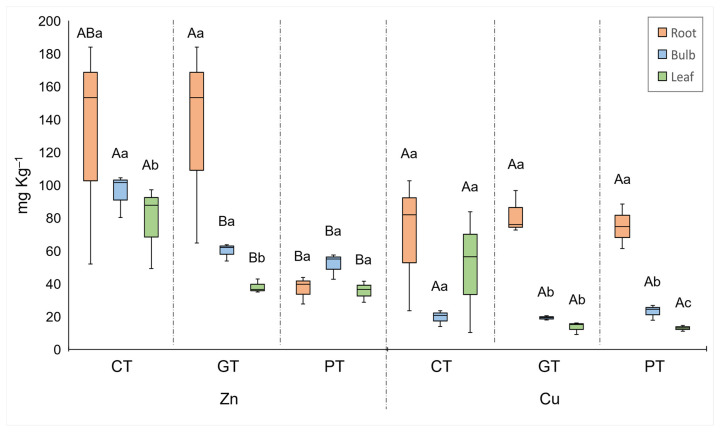
Median, quartiles, and range of Zn and Cu concentrations in different plant organs. Treatments: CT = control, GT = gold tailings, and PT = platinum tailings. Different capital letters indicate a significant difference among treatments for the same organ (*p* < 0.05); different lower-case letters indicate a significant difference among organs within the same treatment (*p* < 0.05).

**Figure 7:**
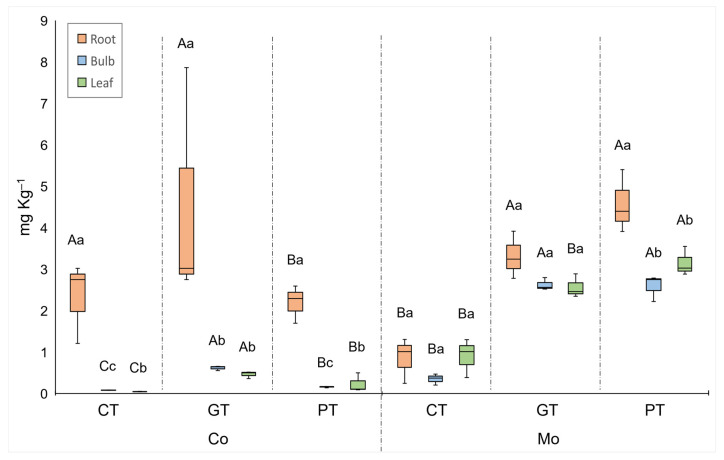
Median, quartiles, and range of Co and Mo concentrations in different plant organs. Treatments: CT = control, GT = gold tailings, and PT = platinum tailings. Different capital letters indicate a significant difference among treatments for the same organ (*p* < 0.05); different small letters indicate a significant difference among organs within the same treatment (*p* < 0.05).

A PCA (Figure 4) indicated the key PTMs that were associated with the plant organs of each soil treatment. Arsenic, Co, and Cu showed a stronger relationship with the root concentrations of *C. bulbispermum* grown on the gold tailings. Chromium, Cd, Mo, and Ni concentrations were highest for the roots of plants grown on the platinum tailings. Zink had the highest concentrations in the roots of the control. A grouping of bulbs and leaves was evident (Figure 4), showing no increased concentrations in the trace elements in the leaf organs across the treatments. The concentrations of PTMs were generally higher in the plant roots compared to the bulbs and leaves across all the treatments (Figure 5, Figure 6 and Figure 7).

Cadmium and Mo concentrations of the plant organs were significantly higher for the plants grown in the tailings compared to the control treatment (Figure 5 and Figure 7). The Cd concentrations in the roots from the platinum tailings were significantly higher than those in the gold tailings. Cobalt, Cd, and Mo showed the highest accumulation in the roots from the tailings (Figure 5 and Figure 7), and Zn was highest in the control and in the gold tailings, while Cu had similar concentrations in the roots of all the treatments (Figure 6).

**Figure 8:**
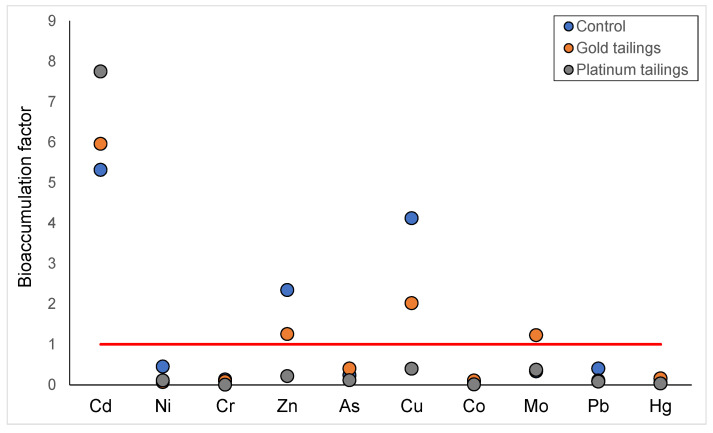
Bioaccumulation factors of PTMs in the roots of *C. bulbispermum*. The red line indicates the bioaccumulation threshold.

A general trend can be seen of decreasing concentrations of PTMs from the roots to the leaves. This indicates little to no translocation of PTMs from the roots through to the bulbs and into the leaves. The concentrations of PTMs were always significantly higher in the roots; therefore, only the root concentrations of PTMs were considered when testing for accumulation. Four PTMs were bioaccumulated (bioaccumulation factor > 1), namely, Cd, Cu, Mo, and Zn (Figure 8). The bioaccumulation of Cd was the highest and accumulated in the roots from all the treatments. The latter three were only accumulated by the roots collected from the gold tailings and the control soil (Figure 8).

## 3. Discussion

### 3.1. What Is the Growth Performance of Crinum Plants on Tailings?

Metal-contaminated soil affects plant growth, specifically the physiological and biochemical processes which result in poor performance and could influence the survival of the plant [21]. Contaminated soil also has an effect on leaf development (e.g., number of leaves and leaf length) and on the further development of plant organs [29]. As would be expected, the plants in the control soil outperformed those grown in the metalliferous tailings. This outcome is supported by another study indicating that plants growing under metal stress conditions experience damaged cell–membrane systems and damage to the structure and function of their organelles [30]. It is evident that the contaminated tailings soils affected the growth of *C. bulbispermum* negatively in the short-term, but the species is hardy and could establish and survive on the tailings.

### 3.2. Do Crinum Plants Take Up Metals When Grown on Tailings?

A range of metals were found at higher concentrations in plant organs grown on tailings compared to the control. Two elements were associated with enhanced or restricted bulb growth. A higher concentration of Mn was associated with larger bulbs and a higher concentration of Mo with smaller bulbs. With bulb size being an indication of plant productivity, this could be explained by Mn being an important micronutrient because of all its vital roles in multiple biogeochemical processes within the plant [31]. Mn supports plant growth and development, regulates and sustains metabolic roles in various cell compartments in the plant, and is an important factor in the oxygen-evolving complex of photosystem II [32,33]. Although Mn supports plant growth, it can also become toxic at high concentrations, leading to chlorosis and reduced growth and photosynthetic capabilities [34]. Plants have, therefore, developed mechanisms to regulate the uptake of Mn, its transport, and storage in the tissue [31].

The higher concentrations of Mo were associated with the reduced size of the bulbs but would not normally be expected to be toxic, because Mo is a micronutrient. It is needed in small quantities, normally between 0.01–0.30 ppm in soil, with Mo concentrations in tissue rarely above 1.5 mg/kg [35]. Mo toxicity is rare and is only expected at very high concentrations. In the case of this study, the mean concentration of Mo in the bulbs was above 2.5 mg/kg, and this elevated level could have had a toxic effect on bulb development. Symptoms of Mo toxicity appear at high concentrations as chlorosis in leaves, and stunted growth was observed in our study [28,36].

### 3.3. Which Organs (Roots, Bulbs, or Leaves) Take Up PTMs?

Tailings are rich in PTMs such as As, Cd, Cr, Cu, Hg, Ni, Pb, and Zn [9]. Most of these were evident for the tailings in this study and, subsequently, in the tissue of *C. bulbispermum*. Our study indicated that these PTMs were not translocated to the leaves and bulbs and were predominantly found in the roots, indicating limited translocation of PTMs. Five PTMs were found at higher concentrations in the roots than in other plant organs, namely, Cd, Co, Cu, Mo, and Zn. Limited translocation of these PTMs occurred; thus, we can assume this species is an excluder of PTMs [37].

The lack of translocation of PTMs and their elevated concentrations in the roots indicate a mechanism actively hindering translocation and storage in the bulb or above-ground organs. This could include detoxification mechanisms such as the induction of antioxidative systems [38], repairing the cell membranes damaged by the PTMs [39], an upregulation of photosynthetic systems [40], enhanced uptake of more essential and nutritious elements [41], the redistribution of PTMs [42], and the regulation of plant metabolism mechanisms [43].

It is reported [44] that compartmentalization can remove toxic elements through the synthesis of metal-binding proteins. Other studies indicate that detoxification can occur through the production and active participation of osmolytes in plants, including proline, glycine, betadine, and sugars [45]. The blocking of PTM translocation to the leaves limits contamination and makes *C. bulbispermum* safe to use as a medicinal plant. This implies that *C. bulbispermum* cannot be used for phytoremediation or phytoextraction but is a strong contender for phytostabilization. The importance of this species lies in its large underground storage organ and traditional/medicinal value. The genus has produced more than 170 different medicinal compounds, of which most are alkaloids [46]. Our finding that it does not accumulate PTMs is, therefore, significant to this value.

### 3.4. Key PTMs Associated with the Plant Organs and Each Treatment

The highest concentrations of Cd were recorded for the roots from the platinum tailings. This was expected [47], as increasing concentrations of Cd in the soil surrounding plant roots result in higher uptake. Higher concentrations of Cd in the rhizosphere resulted in shorter roots [41] and a change in root anatomy (deformation). This was observed for plants grown in the platinum tailings. Cadmium is common in platinum tailings, but Zn is a common element found in gold mine tailings [48]. Plants grown on the gold tailings and in the control treatment showed a high concentration of Zn in the roots, possibly as a result of the lower pH [48]. Zink is important for plant growth and for the development of root structures as well as enzyme and chlorophyll production [49]. Zink becomes toxic at concentrations above 0.1 mM and has toxic effects on plant development. These effects depend on the plant genotype, exposure, and external bioavailable concentration. The most common effects are the inhibition of growth, chlorosis, and cell death [50], but these were not observed in this study.

For plants to function and develop properly, certain quantities and ratios of minerals are needed. Amongst these, Cu is vital to several physiological and biochemical processes in plants [51]. When Cu levels are at an optimum, it is regarded as a crucial micronutrient for all organisms. In plants, Cu has many functions, from mitochondrial respiration to oxidative stress response and hormone signaling [51]. But the opposite is also possible, when Cu is above optimal levels it can destabilize membrane activity, affect photosynthesis, and alter enzyme activity, resulting in growth inhibition [52]. We found higher concentrations of Cu in the roots from the tailings, but the control treatment did indicate comparatively more Cu uptake in the leaves that could be the result of the better bioavailability of Cu in the control soil, with its much lower pH [53]. No Cu toxicity was detected, and the plants in the control treatment outperformed those in the tailing treatments.

Cobalt is considered a beneficial element for plant growth but can be toxic at elevated levels [54]. It is also needed by microorganisms for N fixation, and its deficiency can negatively influence the production of N and negatively impact the growth of the plant [55]. The symptoms of Co deficiency are similar to N deficiency, namely, necrosis, leaf chlorosis, and growth delay [56], as was observed for the plants grown in the tailings.

### 3.5. Bioaccumulation

Bioaccumulation of Cu, Cd, Mo, and Zn occurred in plant roots. The bioaccumulation of certain elements can be expected if they are beneficial to the growth and development of the plant. Copper and Zn are both beneficial to plant development [57] and were bioaccumulated in the roots in the control treatment. As the control plants outperformed those grown in the tailings, this indicated that these PTMs were beneficial to the plants and that the bioavailability of these elements was sufficient for uptake. The accumulation of Mo was highest for the plants in the gold tailings, even though the Mo concentrations among the soil treatments did not differ. As discussed earlier, this could lead to toxicity and can negatively affect the growth of the plants, as observed. Bioaccumulation of Cd was highest in the platinum tailings, reflecting the higher levels in the soil. Cadmium is not needed by plants but is known to accumulate in plant tissue and affects the regulation of Zn and Fe, which are important micronutrients [58]. Cadmium also causes morphological, physiochemical, and structural changes in plants, which can cause chlorosis and the inhibition of lateral root formation and stomal density [59]. The bioaccumulation of certain elements in the roots of *C. bulbispermum* adds to the idea that it can be used as a phytostabilizer in restoration, because it keeps the contaminants in the vadose zone and prevents off-site contamination [17].

## 4. Materials and Methods

### 4.1. Focal Species

*Crinum bulbispermum* was chosen for this study because it is widespread in the Highveld of South Africa. It is a bulbous geophyte that lies dormant over winter, surviving with a large (up to 9 kg) underground storage organ which is protected from frost, drought, grazing, and fire [60,61]. Moreover, it has the added advantage of being a carbon sequester, considering its large underground storage organ, and its contracting roots prevent soil erosion [60]. It is also a widely used medicinal plant [46]. It is easy to cultivate from its calcitrant seeds [62].

### 4.2. Pot Trials

A greenhouse experiment was performed to assess the effect of different toxic soils on the growth of *C. bulbispermum*. There were three soil treatments, namely, commercial standard potting mix (control), gold tailings, and platinum tailings. The potting mix consisted of 50% topsoil and 50% organic matter. Ten seeds were sown per pot (10 L; 25 × 22 cm) and thinned to six seedlings and six pots per treatment (*n* = 6 seedlings × 6 pots × 3 treatments = 36). The pots were arranged completely randomized in the greenhouse. The day and night temperatures were set to 25 °C, subjected to photosynthetic active radiation ranging between 600 and 800 mol m^−^^2^ s^−^^1^, and the pots were drenched with reverse osmosis-treated water every two weeks for one year, excluding the period from winter dieback to re-emergence (June–August).

### 4.3. Measurements

The plants were grown for 12 months. Plant growth was measured monthly to assess the condition of the seedlings: (1) the bulb width (mm) at end of the experiment, (2) the leaf count monthly, (3) the leaf length monthly, measured from the base to the apex of the leaves (mm), and (4) the mortality by end of the experiment.

Plant tissue was also chemically analyzed after 12 months to determine the uptake of PTMs from the respective soil treatments. The plants from each of the treatments were collected (*n* = 3 plants × 3 treatments = 9 plants), weighed, and separated into roots, leaves, and bulbs and then dried. The dried material was rinsed in distilled water, washed in a 1% HCl solution to remove dust and soil, and rinsed again in distilled water. All the material was dried at 40 °C for seven days. The dry material was analysed by ICP-MS to determine the trace metal content.

### 4.4. Statistical Analyses

#### 4.4.1. Plant Growth

The bulb width, leaf count, and leaf length were evaluated individually in univariate one-way permutational multivariate analysis of variance (Permanova) designs, given the non-normality of the data (Shapiro–Wilk’s, *p* < 0.05). The factor was the “soil treatment” (fixed, three levels: control soil, gold tailings, and platinum tailings). A PCA with the same variables and factors was performed for the visualization of possible groupings and main variables.

#### 4.4.2. Bioaccumulation

Arsenic, Cd, Co, Cr, Cu, Hg, Mn, Mo, Ni, Pb, Sr, and Zn concentrations in the roots, bulbs, and leaves were compared between plants from the different treatments in uni- and multivariate analyses. A distance-based linear model (distLM) was performed from a Euclidean-distance matrix with the developmental variables from the study plants. Firstly, the trace elements in the different organs were used as predictive variables to assess the influence of PTMs on plant development. A dbRDA was performed to investigate the relationship between metal concentrations and plant organ development. Secondly, the element concentrations in the plant organs were used in a PCA, in search for the main elements (i.e., those with higher correlation with the principal components) as possible drivers of the distribution of the data. Thereafter, the soil treatment factor was tested for differences in each main trace element concentration, in the different plant organs. This was carried out in a univariate two-way Permanova design with “soil contamination” (fixed, three levels: control, gold tailings, and platinum tailings) and “plant organ” (fixed, three levels: roots, bulbs, and leaves) as factors. Finally, the bioaccumulation factors [55] between the roots and the soil were calculated for each of the main elements.

## 5. Conclusions

Potentially toxic metals in the tailings negatively affected the development and growth of *C. bulbispermum* in terms of leaf length and bulb width when compared to the control. Although the growth was hampered, *C. bulbispermum* could survive on the tailings. The plants on the tailings were affected by metal toxicity, but this was presumed to be restricted to the root zone, as limited translocation of elements occurred from the roots to the leaves. Subsequently, no PTMs were available for storage in the bulbs or leaves. A literature review indicated particular PTMs to aid growth and development, and others were identified as possibly negatively affecting *C. bulbispermum*.

In this study, the bioaccumulation of Cd, Cu, and Zn was recorded in the roots. Cadmium bioaccumulation was a phenomenon in the tailings, but Cu and Zn bioaccumulation was not restricted to the plants grown on the tailings. Importantly, the plant roots did not translocate PTMs, in either the stressed or control treatments. Our results indicate that *C. bulbispermum* may be grown in metalliferous tailings and remain safe to use by local communities for medicinal purposes as the PTM concentrations are at acceptable levels. This needs to be monitored, and, on gold tailings specifically, attention should be given to the metalloid As, of which the highest concentrations of 30.01 and 14.3 mg/kg were recorded in the tailings and plant tissue, respectively. This study indicates that *C. bulbispermum* is not a hyperaccumulator and that it is not recommended for phytoextraction. However, it still shows resilience, although root growth is hampered, and can be used for restoration purposes involving phytostabilization. Considering its performance on the control soil, *C. bulbispermum* can be introduced into future restoration plans if the tailings are ameliorated. The results still need to be confirmed in less controlled conditions on tailings dams.

## Figures and Tables

**Table 1:** Nutrient status, pH, and potentially toxic metal(loids) (PTMs) (mg/kg) of the different soil treatments.

Sample	Ca	Mg	K	Na	Cd	Cu	Co	Mo	Mn	Zn	pH (H_2_O)
Control	2601.6	912.7	1771.9	848.9	0.0451	16.83	32.41	2.566	442.1	55.32	5.71
Gold tailings	1572.5	46.3	22.2	7.9	0.1051	40.45	85.65	2.697	270.8	51.62	6.70
Platinum tailings	261.0	35.1	13.2	6.9	0.1237	188.5	285.4	2.579	2131	170.1	7.21

## Data Availability

The data presented in this study are available on request from the corresponding author. The data are part of a Masters degree study and will not be publicly available until the dissertation is published. All dissertations and theses of the North-West University are available at https://library.nwu.ac.za/theses-and-dissertations (accessed on 13 September 2023).
